# Variants in *BRCA1/2* in a hospital-based cohort in Chile and national literature review

**DOI:** 10.3332/ecancer.2024.1683

**Published:** 2024-03-21

**Authors:** Fernanda J Martin, Isabel M Saffie, Mabel A Hurtado, Diana Avila-Jaque, Rodrigo A Lagos, Carolina A Selman, Jonathan Z Huserman, Valentina A Castillo, Badir J Chahuán

**Affiliations:** 1 Unidad Asesoramiento Genético Oncológico, Fundación Arturo López Pérez, Santiago 7500921, Chile; 2 Cirugía de mama, Fundación Arturo López Pérez, Santiago 7500921, Chile; 3 Sección de Genética, Hospital San Juan de Dios, Santiago 8350488, Chile; 4 Unidad estadística, Fundación Arturo López Pérez, Santiago 7500921, Chile; 5 Subdirección Unidades Diagnósticas, Fundación Arturo López Pérez, Santiago 7500921, Chile; 6 Departamento Genética, Hospital Base San José Osorno, Osorno 5311523, Chile; 7 Departamento Genética, Hospital Clínico Universidad de Chile, Santiago 8380453, Chile; 8 Departamento Genética, Hospital Dr. Sótero del Río, Santiago 8150000, Chile; a https://orcid.org/0000-0002-7167-8850; b https://orcid.org/0000-0002-4723-5750; c https://orcid.org/0009-0002-7787-6847; d https://orcid.org/0000-0002-5806-6227; e https://orcid.org/0000-0002-9355-3282; f https://orcid.org/0000-0003-3133-6706

**Keywords:** BRCA1, BRCA2, germline variants, Chile

## Abstract

**Purpose:**

The aim was to assess the diagnostic yield of next generation sequencing (NGS) multi-gene panels for breast and ovarian cancer in a high-complexity cancer centre in Chile. Additionally, our goal was to broaden the genotypic spectrum of BRCA variants already identified in Chilean families.

**Methods:**

Retrospective analysis was conducted on the genetic test results of 722 individuals from Fundación Arturo López Pérez’s genetic counselling unit between 2016 and 2021. A comprehensive literature review encompassing articles analysing the frequency of germinal pathogenic variants in *BRCA1/2* within the Chilean population was undertaken.

**Results:**

23.5% of the panels had positive results, with 60% due to pathogenic variants in the *BRCA1/2* genes. Seven previously unreported variants in *BRCA1* from Chilean studies were identified.

One or more variants of uncertain significance were detected in 31% of the results, and 11.5% of the families in this cohort presented copy number variants (CNVs) in *BRCA1/2*.

8 studies analysed the frequency of pathogenic variants in *BRCA1/2* in the Chilean population between 2006 and 2023, with a frequency between 7.1% and 17.1%.

51 *BRCA1* variants in 149 families have been reported in Chile and 38 *BRCA2* variants in 132 families. Nine founder pathogenic variants identified by one study were present in 51.9% of the total Chilean families reported.

**Conclusion:**

Our findings advocate for the integration of NGS multi-gene panel testing as a primary strategy within our population. This approach allows for the comprehensive assessment of single nucleotide variants and CNVs in *BRCA1/2*, alongside other high and moderately penetrant genes associated with breast and ovarian cancer.

## Introduction

Identifying individuals with hereditary susceptibility to cancer has been a crucial aspect of cancer care acknowledged by the American Society of Clinical Oncology since the 1990s [1]. However, access to genetic counselling and testing remains unequal across ethnic groups and is significantly linked to a country’s level of development [2, 3]. Presently in Chile, genetic testing coverage within health insurance schemes is diverse, particularly limited for sequencing studies. Consequently, its utilisation is inconsistent and heavily reliant on patients’ socioeconomic capabilities [4].

Prior investigations in Chile among individuals with a personal or familial history of breast and ovarian cancer have revealed varying frequencies of pathogenic (P) variants in *BRCA1/2*, ranging between 7% and 17% [5–14]. One study identified a notable frequency of nine variants identified as founders using haplotype analysis [6]. Our institution has an oncology genetic counselling unit that focuses on high-risk patients for hereditary cancer, maintaining a registry of germline studies since 2016. Through the analysis of this cohort, our aim was to ascertain the diagnostic yield of genetic tests for breast and ovarian cancer within a high-complexity cancer centre in Chile. Furthermore, our objective was to broaden the spectrum of genotypic variants previously identified in *BRCA1/2* within Chilean families. This endeavour aims to enrich the genomic understanding of our population and foster the development of strategies that positively impact the health of our patients, their families, and the community.

## Methods

We retrospectively analysed the results of the genetic tests of 722 individuals from the genetic counselling unit of Fundación Arturo López Pérez (FALP) between May 2016 and December 2021. Annually, Chile reports approximately 5,331 cases of breast cancer, according to GLOBOCAN, with our institution treating around 400–500 of these cases, constituting roughly 10% of the country’s total cases.

During this period, the unit primarily cared for patients with breast and ovarian cancer, either self-referred or via referrals from other specialists based on their personal and/or family cancer history. The genetic counselling structure involved three primary components: 1) Consultation with a nurse to gather background information and create a three-generational family tree, 2) Pre-test consultation with a genetic counsellor (breast surgeons trained in oncological genetic counselling), and 3) Post-test consultation to deliver results and discuss implications. The test indication was determined based on the evolving criteria of the National Comprehensive Cancer Network.

All reported studies were done by next-generation sequencing (NGS) with copy number variant (CNV) analysis performed by the laboratory Invitae^®^ using Illumina technology. Genomic DNA obtained from peripheral blood or saliva samples was analysed, encompassing coding regions of genes, 20 bp of intronic flanking regions, and specific genomic regions of interest. Laboratory-reported analytical sensitivity and specificity was >99% for single nucleotide variants (SNVs), <15 bp insertions and deletions, as well as exon-level deletions and duplications.

The results were compared to the GRCh37 reference genome. Variant classification was performed using the laboratory’s SHERLOC system following the guidelines established by the American College of Medical Genetics and Genomics and the Association for Molecular Pathology for genetic variant classification [15].

Bivariate statistical analysis was performed in a subgroup of female breast cancer patients to evaluate the relationship between the dichotomized response variable (positive or negative test result) and the variables: age, age <50 years, triple-negative breast cancer, ≥2 primary cancers, positive family history of cancer, known family variant, oestrogen receptor (ER) status, progesterone receptor (PR) status, HER2 status. Results with Pathogenic/Likely pathogenic (P/LP) variants with or without variants of uncertain significance (VUS) were considered positive, and results with VUS-only or no variants were considered negative.

The Kruskal–Wallis test was utilised for continuous variables due to unmet normality and homoscedasticity assumptions. Fisher’s exact test examined relationships between categorical variables. A logistic regression model was performed for the response variable result of the test, and the odds ratio coefficients, the 95% confidence intervals, and the corresponding *p*-value were calculated for each predictor variable.

A literature search was carried out through Pubmed, SciELO, LILACS, and Google Scholar using combinations of the terms ‘CHILE’, ‘CHILEAN’, ‘BRCA’, ‘GERMINAL’, ‘GERMLINE’, in English and Spanish, with no date limit. The articles that analysed the frequency of germinal P variants in *BRCA1/2* in the Chilean population were selected.

## Results

During the analysed period, among 891 indicated tests, 722 were conducted primarily on women (91.1%) and patients diagnosed with cancer (75.2%). Of those diagnosed with cancer, 96% were female, with 92.2% having breast and/or ovarian cancer. Multigene panel studies for breast and ovarian cancer accounted for 56.2% of cases (7–36 genes), followed by multicancer panel requests in 22.9% (47–84 genes), and single variant or single gene studies (colon, thyroid, gastric, among others) in 20.7% of cases. Cascade studies of family variants constituted 17.5% of all genetic tests, with a positive result rate of 47.2% (Table 1).

**Figure 1.**
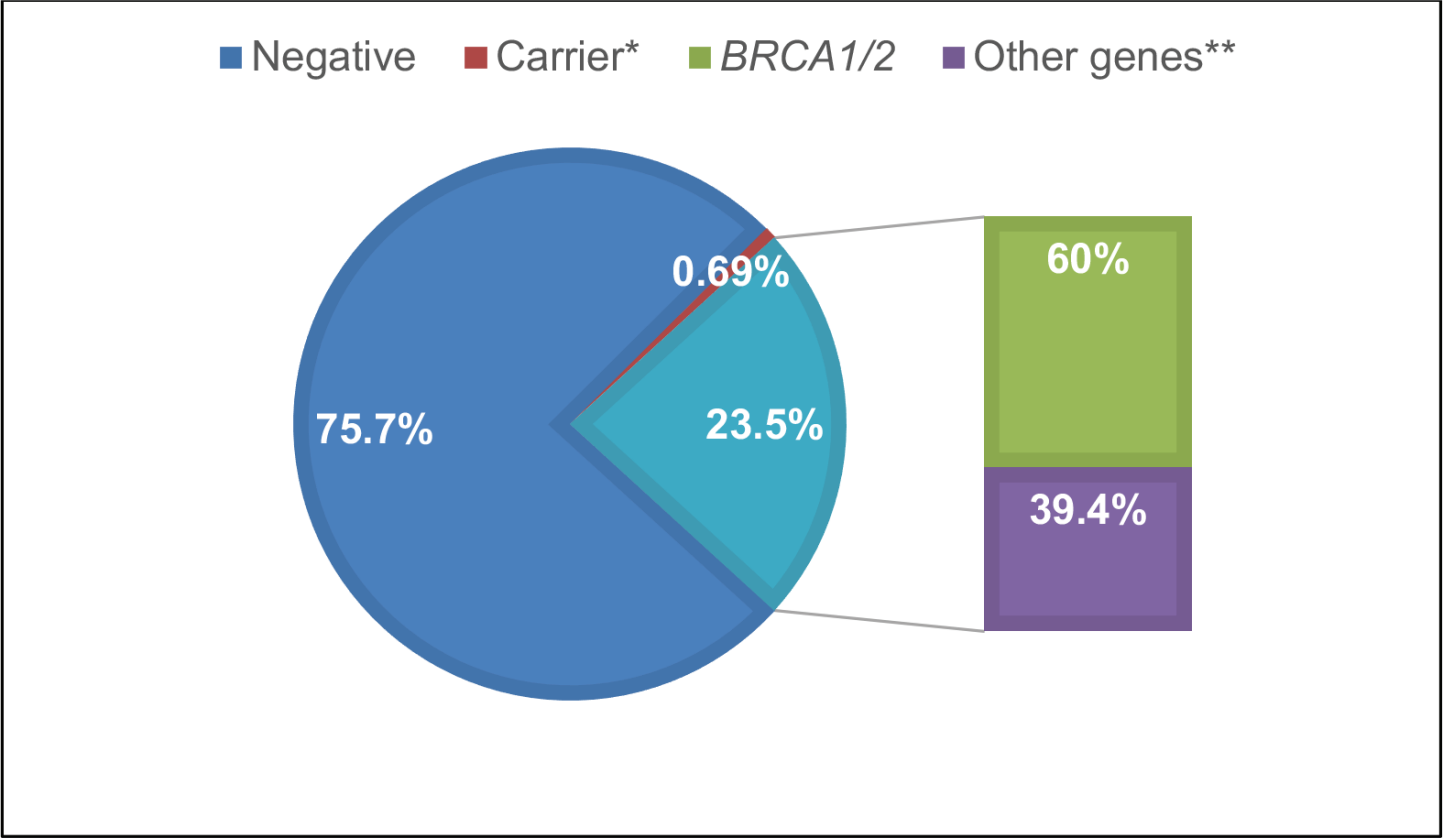
Test results FALP study cohort. *Carrier: PV found in a gene associated with an autosomal recessive condition. **Other genes: PV in cancer predisposition genes other than BRCA1/2.

Overall, 23.5% (170 individuals) of panels yielded positive results, with 60% (103 individuals) due to P or LP variants in the *BRCA1/2* genes (Figure 1). Additionally, 39.4% of cases (67 individuals) exhibited P/LP variants in other genes: *APC, ATM, ATR, AXIN2, CDKN2A, CHEK2, FANCM, MSH2, MSH6, NBN, NF1, PALB2, PTEN, RAD50, RAD51c, RAD51D, RET,* and *TP53* (see Supplementary Table 1; all supplementary material can be found at https://figshare.com/articles/dataset/Supplementary_material_docx/24942498). Five P variants in genes with an autosomal recessive inheritance mode were regarded as carrier results.

Of the identified *BRCA* variants, 53 individuals from 31 families had P variants in *BRCA1*, while 50 individuals from 30 families had P variants in *BRCA2*. The vast majority (85.4%) with* BRCA*1/2 variants were women, among whom 63% had cancer. Specifically, 75.3% of *BRCA*-positive individuals with cancer had breast cancer, 13.8% had ovarian cancer, and 6.1% had both breast and ovarian cancer. Only 2 individuals (3%) presented cancer outside the *BRCA1/2* spectrum, with testicular cancer. Both men were studied for the same familial variant (*BRCA2*: c.6275_6276delTT) identified in a female index case with breast cancer (Table 2).

Moreover, seven new variants previously unreported in *BRCA1* in Chilean studies were identified, while all variants in *BRCA2* had been previously reported (Table 3). The majority of these variants, except for one missense variant (*BRCA1*: c.5095C>T) were truncating variants (nonsense or frameshift). 11.5% of the families in this cohort (7 families) presented CNVs. Three families presented a pathogenic deletion of exon 12; one presented a deletion of exons 13–15, and three showed a pathogenic duplication of exons 3–7 (Table 3).

Furthermore, 38.8% of positive results for *BRCA1/2* variants were from family cascade tests. Relatives up to third degree were tested in 16 families, averaging 2.8 individuals per index case with a positivity rate of 46.6%.

One or more VUS were identified in 31% of the results (224); from these, in 84.8% (190 individuals), only one or more VUS were identified, and in 15.1% (34 individuals), a P/LP variant was identified in conjunction with a VUS. In total, 268 VUS were recognised in 63 genes. 5 VUS were identified in *BRCA1* and 14 VUS in *BRCA2* (Supplementary Table 2).

Bivariate statistical analysis highlighted a significant difference between a positive and negative test result for individuals with ≥2 primary cancers, a positive family history, and a previously known family variant. However, age, age <50 years, triple-negative breast cancer, ER, PR, and HER2 status showed no statistically significant differences (Supplementary Table 3). A logistic regression model did not yield any statistically significant associations (Supplementary Table 4). To compare the results (positive versus negative as defined above) of the multicancer panel with the breast and ovarian multigene panel, a two-sample test for equality of proportions with continuity correction was used, resulting in a *p*-value of 0.1026 and a chi-square of 2.6640. Hence, there was no statistically significant difference between them.

In the literature review, from 2006 to 2023, eight studies analysed the frequency of LP/P variants in *BRCA1/2* in the Chilean population with cohorts of variable size, inclusion criteria, and technology used for testing [5–8, 12–14, 16] (Table 4). One study [9] was excluded due to variant nomenclature discrepancies, and because the P variants reported were now classified as benign or VUS. The frequency of LP/P variants in *BRCA1/2* across studies using Sanger or NGS (all except Sanchez *et al* [12]) was between 7.1% and 17.1%. The study conducted by Alvarez *et al* [16] exclusively focused on analysing the frequency of nine previously described variants in *BRCA1/2* from a prior study. A partial overlap was observed between the individuals reported in the Ossa Gomez *et al* [13] cohort and those in our study due to all examinations from our institution being referred to the Invitae laboratory. This temporal overlap occurred between 2016 and 2019 in both studies, hence, individuals were only considered once for the analysis. To avoid redundancy and ensure an accurate representation, we limited our analysis to one individual per family for each variant in this cohort. Similarly, this approach was applied across all studies with adequate information to distinguish index patients from cascade testing, thereby preventing potential overrepresentation of variants. Another study [14] carried out between 2012 and 2021 also utilised Invitae’s services for tests. However, this study did not provide specific variant details for positive cases. Consequently, our analysis considered solely the overall positive test rate from this particular study.

In Chile, 51 *BRCA1* variants have been reported among 149 families and 38 *BRCA2* variants among 132 families. Most of these variants have been observed in other Latin American populations or globally. However, two *BRCA1* variants (c.187_188insA, c.3710_3711del) and three *BRCA2* variants (c.-39-?_425+ ?del, c.7397dup, c.8223_8224dup) remain unreported in databases such as ClinVar, human gene mutation database, databases registered in latingen.org (Mexico, Brazil, and Argentina), or in any other published articles to date. Notably, 62.9% (56 variants) were found in single individuals (Supplementary Table 5).

Nine founder variants identified by Alvarez *et al* [6] in 2017, highlighted in Table 3, were present in 50.8% of the results of this cohort. Considering the total previously reported Chilean families [5–8, 10, 12, 13] along with this cohort, 51.9% carried one of these variants. It’s important to note that 60 families were exclusively studied for these nine variants [6, 16] (Supplementary Table 5).

## Discussion

Our FALP cohort demonstrated a diagnostic yield of 23.5%, akin to recent panel testing studies by Acevedo *et al* [14], surpassing earlier Chilean reports. This increase was probably due to patient selection criteria and multi-gene panel testing. LP/P variants in *BRCA1/2* genes (around 14%) were consistent with recent studies in Chilean and Latin American populations [5, 6, 13, 17], surpassing international rates of 5%–10% in women from more developed countries [17–22]. A review encompassing 33 studies across 13 Central and South American countries in 2015, involving 4,835 individuals, showcased a broad spectrum in *BRCA* P variant prevalence. Results varied significantly, spanning from 1.2% to 47.8%. However, within unselected breast cancer cohorts, the prevalence ranged between 1.2% and 4.9%, aligning with rates observed in non-Hispanic populations [23]. In that review and the present one, the lack of homogeneity in the selection criteria, population size, and testing methodologies from the different studies could account for some differences in the prevalence of *BRCA* variants. The limited access to genetic counselling and studies in the region, as well as the lack of coverage of therapeutic, risk reduction, and early detection interventions that have shown cost-effectiveness in people with *BRCA1/2* variants, all factors that discourage testing [5, 13, 16, 24], likely contributed to a selection bias towards patients more likely to test positive. Alternate hypotheses suggest that individuals of Latin American or African ancestry might exhibit a higher prevalence of P variants. Additionally, the region’s higher occurrence of triple-negative breast cancer and cases at younger ages might drive more targeted testing practices [24]. While Chile and other Latin American countries demonstrate an average inbreeding coefficient linked to consanguinity higher than that of West European countries and North America but considerably lower than African and Middle Eastern countries [25], the precise relationship between the frequency of *BRCA* carriers and this coefficient remains unclear. This complexity is further compounded by varying colonisation histories and genetic admixture across American countries, resulting in distinct genetic compositions and *BRCA* variant spectrums [10]. Estimates indicate that approximately 10.4% of *BRCA* P variants are shared between Hispanics in the United States and Latin American populations. Within Latin America and the Caribbean, about 8.2% of reported *BRCA* variants were identified in more than one country [23]. Despite 94.3% of the identified variants having prior records in other countries, most were not shared among families in this cohort. Intriguingly, some variants were exclusively reported outside the Americas, in regions such as China, Korea, Singapore, Australia, Palestine and several European countries (Supplementary Table 5).

There’s a scarcity of data on population-specific risks and inadequate systematic studies on the prevalence of genetic variants associated with breast cancer across Latin American populations. This reflects considerable heterogeneity among countries in the region, emphasising the necessity to consider these differences when implementing precision medicine initiatives. Nations exhibiting a high prevalence of *BRCA* P variants might derive considerable benefits from adopting more aggressive testing strategies. Conversely, employing recurrent variant panels for testing could offer a cost-effective solution to enhance genetic testing in certain countries, although this might not be universally applicable across all regions.

The statistical analysis undertaken in this study revealed a lack of a straightforward correlation between various patient selection criteria and a positive test result. Notably, there were contradictory results between the bivariate analysis and the regression model. These discrepancies might stem from the limitation of bivariate analysis, which doesn’t account for other potentially influential variables related to those of interest. Sample size could also have influenced these findings. It’s crucial to acknowledge the limitations inherent in these statistical techniques within this study context, alongside considering the NCCN selection criteria filter applied in this specific cohort. Consequently, these results cannot be extrapolated to the general population due to the selection bias in this dataset.

Up to this point, research studies in Chile have primarily cantered around hospitals and selected populations at a heightened risk of presenting genetic variants. This focus has led to a notable gender bias in cohorts of *BRCA*-mutated individuals in Chile, primarily among women with breast and ovarian cancer. The absence of population-based studies and limited research on cancers beyond breast and ovarian have contributed to this skew in the data.

Estimates of the carrier rate for *BRCA1/2* variants in the general population, from various international studies [17, 24, 26–28], range between 0.1% and 0.38%. Considering Chile’s population of approximately 17,500,000 inhabitants (www.censo2017.cl/), this translates to an estimated count of carriers ranging between 17,500 and 66,500 individuals within the country. This highlights the potential presence of undiagnosed carriers in the population, indicating a need for more comprehensive, population-focused research to understand the prevalence of these variants across various demographics and cancer types beyond breast and ovarian.

The findings from Alvarez *et al* [6] initially suggested a high frequency (77.5%) of nine identified variants, which were determined as founders through short tandem repeat minimal haplotype analysis in a cohort of 453 individuals. However, subsequent studies [5], including the analysis conducted here, observed a lower frequency (51.9%) for these variants reported in Chilean individuals. This discrepancy underscores the need for a more comprehensive approach to genetic testing, especially concerning the identified founder variants.

While the use of panels focusing on these founder variants has been proposed as a cost-effective screening method in Chile [6, 16], it’s essential to recognise its limitations. Such a strategy may overlook the identification of other SNVs or CNVs in *BRCA1/2*, which, as per this study, are present in approximately half of the cases in Chile. Moreover, restricting testing to these founder variants would also limit the detection of variations in other genes like *PALB2, TP53, ATM,* and *CHEK2,* among others, which represent a significant proportion of cases in this and other studies [5, 13, 14] and have established guidelines for management, risk reduction, and early detection strategies [29]. Considering the declining costs of NGS and the demonstrated efficacy of multigene extended panels in the Latin American population [13], a shift towards NGS-based multigene testing is warranted. This approach provides a more comprehensive evaluation of various genes associated with hereditary cancer, especially in populations whose genetic landscape remains incompletely understood, such as in Chile. Alternatively, a staggered testing approach involving the initial screening with the RT-PCR protocol developed by Alvarez *et al* [16] followed by NGS testing in patients with a negative initial study, could be considered [16]. However, evaluating the cost-effectiveness of such a strategy would necessitate assessing the impact of delayed actionable molecular diagnoses in patients lacking the identified founder variants, alongside the expenses associated with double testing in a significant proportion of patients.

The utilisation of multigene panels raises concerns regarding the detection of VUS. These variants can pose challenges by increasing the workload for clinicians in interpreting test results, potentially leading to misinterpretation, and even causing a negative psychological impact on patients [30]. In populations outside of European or non-Hispanic white groups, such as in Latin America, the incidence of VUS tends to be higher [30]. However, this does not seem to significantly affect the positivity rate of the studies. Additionally, it’s reassuring to note that most of these variants eventually get reclassified as benign [13]. An optimistic aspect is the growing number of individuals undergoing testing, leading to the establishment of local databases that document variants identified within specific populations. This accumulation of data will play a crucial role in facilitating the interpretation of results over time.

The significant percentage of positive results for *BRCA1/2* variants (38.8%) within families of this cohort highlights the effectiveness of cascade testing (Table 2). This testing approach has demonstrated its cost-effectiveness by enabling individuals without a history of cancer to recognise their heightened risk for hereditary conditions. It serves as a means to implement preventive measures aimed at reducing risk and facilitating early detection of cancer in those at an increased predisposition due to familial genetic factors [14, 31].

The implementation of genetic counselling for cancer patients with risk factors, as mandated by Chile’s National Cancer Law (www.bcn.cl), faces substantial challenges in practice. Recent studies in Chile [14] reveal that despite clear indications for germline testing, less than 20% of eligible patients undergo this counselling. Several obstacles contribute to this situation, including a shortage of healthcare professionals trained in genetic counselling, limited coverage for most molecular testing within the public health system, disparities in coverage across the private system, lack of public awareness, and insufficient medical referrals [13, 14, 4]. Overcoming these multifaceted challenges is crucial to improving access to genetic counselling and testing for at-risk individuals in Chile.

The inadequate coverage of genetic testing creates missed opportunities to implement prevention guidelines and risk-reducing interventions, particularly for high-risk individuals. Data from Chile’s national health survey reveals concerning statistics regarding mammography screenings, indicating that only 33% and 53% of women over 50 years underwent mammograms within the last year in the public and private healthcare systems, respectively [32]. This represents a significant challenge in prevention, especially for those high-risk women unaware of it.

Prophylactic oophorectomy can reduce the risk of ovarian cancer by 96% and breast cancer by 68% in *BRCA*-mutated women [33, 34]. Similarly, bilateral prophylactic mastectomy offers an 87% risk reduction for breast cancer in women without a history of cancer and reduces the risk of contralateral breast cancer by 97% in those with previous breast cancer [35, 36]. Surgery is generally performed subcutaneously, prioritising immediate reconstruction to restore the patient’s anatomy, thereby lessening the psychosocial impact of the procedure.

Moreover, knowing a patient’s *BRCA* mutational status provides opportunities for targeted pharmacological interventions. In metastatic HER2-negative breast cancer patients with *BRCA* mutations, Poly(adenosine diphosphate–ribose) polymerase (PARP) inhibitors have shown significant benefits, offering an effective therapeutic approach [37]. Additionally, in high-risk HER2 negative early breast cancer patients with *BRCA* mutations, PARP inhibitors have been explored as an adjuvant treatment option [38]. These advancements underscore the importance of genetic testing for targeted therapeutic strategies, optimising treatment outcomes in patients with *BRCA* mutations.

Indeed, this study’s focus on a specific population at high risk for hereditary cancer predisposition can be perceived as both a strength and a limitation. This targeted approach enhances the diagnostic yield for molecular studies within a resource-constrained setting. Including family and cascade testing aligns with the key objectives of cancer genetic counselling, adding value to the study’s comprehensiveness. However, this targeted population might introduce selection bias, limiting the generalisability of findings to broader populations. Furthermore, the absence of recorded data on the impact of molecular diagnoses on therapeutic and follow-up decisions represents a notable limitation. Future studies should aim to incorporate these elements to comprehensively assess the clinical implications of genetic testing in guiding treatment strategies and patient management.

## Conclusion

The utilisation of multi-gene NGS panel testing within a specific cohort of individuals at elevated risk for carrying germline variants has demonstrated a high diagnostic yield for identifying P and LP variants in high and moderate-risk breast and ovarian cancer genes. The *BRCA* variant spectrum for our population continues to grow with broader testing strategies. The successful implementation of cascade testing has enabled identifying individuals harbouring *BRCA* mutations, even in the absence of prior cancer history, offering crucial opportunities for preventive measures and risk reduction interventions.

## List of abbreviations

CNV, Copy number variant; ER, Oestrogen receptor; LP, Likely pathogenic; NGS, Next generation sequencing; P, Pathogenic; PARP, poly(adenosine diphosphate–ribose) polymerase; PR, Progesterone receptor; SNV, Single nucleotide variant; VUS, Variant of uncertain significance.

## Conflicts of interest

The authors have no relevant financial or non-financial interests to disclose.

## Funding

This study received no funding.

## Ethical approval

This study was performed in line with the principles of the Declaration of Helsinki. Approval was granted by the Healthcare Ethics Committee of FALP (N° 2022-031-SON-BRS-INT) and carried out in compliance with the current legislation in Chile on Scientific Research in humans of laws 20,120, and 19,628.

## Data availability

The main data analysed during the current study has been included in tables and electronic supplementary material. Further datasets collected and analysed in the study are not publicly available due to confidentiality reasons but are available from the corresponding author upon reasonable request. All newly reported variants in this study were submitted to the ClinVar database (https://www.ncbi.nlm.nih.gov/clinvar/).

## Author contributions

All authors contributed to the study conception and design. Material preparation, data collection and analysis were performed by Fernanda Martin, Diana Avila-Jaque, Isabel Saffie, Rodrigo Lagos, Mabel Hurtado, Valentina Castillo and Jonathan Huserman. The first draft of the manuscript was written by Fernanda Martin, and all authors commented on later versions. All authors read and approved the final manuscript.

## Figures and Tables

**Table 1.** FALP study cohort.

Total cohort (*N* = 722 individuals)			
Sex		Age	
Female	658 (91.1%)	Female	17–78 years (X 43 years)
Male	64 (8.9%)	Male	21–85 years (X 46 years)
Case			
Index case	596 (82.5%)		
Family variant	126 (17.5%)		
Cancer status		Cancer patients	
Cancer (total)	543 (75.2%)	Female	522 (96%)
Non-cancer	171 (23.6%)	Male	21 (4%)
Unknown	8 (1.1%)	Breast/ovarian	501 (92.2%)
		Others^a^	42 (7.8%)
Solicited panel		Results	
Breast/ovary	406 (56.2%)	Negative	547 (75.7%)
Multicancer	166 (22.9%)	Carrier^b^	5 (0.69%)
*BRCA* genes	86 (11.9%)	Total positive	170 (23.5%)
Other panels/genes	64 (8.8%)	*BRCA1*	53 (31.1%)
		*BRCA2*	50 (29.4%)
		Other genes	67 (42.3%)

a Colon, thyroid, prostate, endometrium, gastric, skin, kidney, pancreas, lung, lymphoma

b Pathogenic variant found in a gene associated with an autosomal recessive condition

**Table 2.** *BRCA* FALP cohort.

BRCA cohort (*N* = 103 individuals)			
Sex		Age	
Female	88 (85.4%)	Female	17–78 years (X 40.7 years)
Male	15 (14.5%)	Male	22–76 (X 41.5 years)
Case status			
Index case	63 (61.2%)		
Family variant	40 (38.8%)		
Cancer status		Type of cancer	
Cancer	65 (63%)	Breast	49 (75.38%)
Non cancer	38 (37%)	Ovary	9 (13.84%)
		Breast and Ovary	4 (6.15%)
		Testicular	2 (3,07%)
		Breast-Lung	1 (1.53%)

**Table 3.** P/LP *BRCA* variants FALP cohort.

BRCA1 (NM_007294.3)	Index cases
c.1044T>A (p.Cys348*)	1*
c.1674del (p.Gly559Valfs*13)	1
c.1969C>T (p.Gln657*)	1*
c.2486_2487del (p.Phe829*)	1
c.2960_2964del (p.Lys987Ilefs*3)	1*
c.3331_3334del (p.Gln1111Asnfs*5)	4
c.3485del (p.Asp1162Valfs*48)	1*
c.3756_3759del (p.Ser1253Argfs*10)	1
c.3759dup (p.Lys1254*)	4
c.3817C>T (p.Gln1273*)	4
c.4057_4061del (p.Glu1353*)	1*
c.4065_4068del (p.Asn1355Lysfs*10)	1
c.5095C>T (p.Arg1699Trp)	1*
c.514C>T (p.Gln172*)	1*
c.70_73dup (p.Pro25Leufs*17)	1
Deletion (Exon 12)	3
Duplication (Exons 3-7)	3
c.4358-2323_4968del Partial Deletion (Exons 13-15)	1
Total	31
*BRCA2* (NM_000059.3)	
c.145G>T (p.Glu49*)	2
c.2830A>T (p.Lys944*)	1
c.4740_4741dup (p.Glu1581Valfs*37)	8
c.5146_5149del (p.Tyr1716Lysfs*8)	8
c.5439del (p.Val1814*)	2
c.6024dup (p.Gln2009Alafs*9)	1
c.6220del (p.His2074Thrfs*7)	1
c.6275_6276deITT(p.leu2092Profs*7)	1
c.6445_6446del (p.Ile2149*)	1
c.6468_6469del (p.Gln2157Ilefs*18)	1
c.6727_6728insAT (p.Ser2243Tyrfs*38)	1
c.8068_8069del (p.Val2690Phefs*2)	2
c.8987T>A (p.Leu2996*)	1
Total	30

* Not previously reported in Chilean literature

Variants in bold correspond to founder variants reported by Alvarez *et al* [6]

**Table 4.** Previous Chilean studies.

Author, year	Population	Molecular analysis	% positive for BRCA P/LP variants
Gallardo et al [8]	77 individuals, selection criteria not specified	PCR and SSCP analysis of exons 2–24 of BRCA1, and PPT of exons 10–11 of BRCA2	10.3%
Sanchez *et al* [12]	78 individuals with a family history of cancer and a negative *BRCA1/2* SNV study	MLPA *BRCA1/2*	0%
Gonzalez-Hormazabal *et al* [7]	362 individuals at high risk of breast/ovarian cancer	Complete coding sequence of *BRCA1/2* by conformation-sensitive gel electrophoresis and Sanger sequencing. MLPA in 56 cases with negative SNV study	7.1%
Alvarez *et al* [6]	336 individuals with a personal or family history of breast cancer	147 individuals: amplification of exons 2–24 in *BRCA1* and exons 2–27 in *BRCA2* by PCR, heteroduplex/PTT/SSCP analysis, and Sanger sequencing 306 individuals: NGS (Junior Roche 454) with confirmation by Sanger sequencing.	15.7%
Adaniel *et al* [5]	315 individuals with a personal or family history of breast and ovarian cancer	210 individuals: Sanger sequencing of the entire coding sequence, partial sequencing of selected exons, or sequencing of Jewish-Ashkenazi population founder variants 105 individuals: NGS panel with confirmation by Sanger sequencing or MLPA	17.1%
Ossa Gomez *et al* [13]	1,025 individuals with personal and/or family history of breast/ovarian cancer	Multi-gen panels by NGS (Illumina technology-Invitae)	13%
Alvarez *et al* [16]	60 individuals in a ‘relevant setting’ for genetic screening	RT-PCR of nine founder variants with confirmation by Sanger sequencing	10%
Acevedo *et al* [14]	300 individuals who met NCCN criteria for testing	32 individuals: Sanger *BRCA1/2* 268 individuals: Multi-gen panels by NGS (Illumina technology-Invitae)	12.3%

MLPA: Multiplex Ligation Probe Amplification: SALSA MLPA Kit P002-B1 para *BRCA1* y SALSA MLPA Kit P045-B1 para *BRCA2*

SSCP: single strand conformational polymorphism

PPT: protein truncation test

## Supplementary material

All supplementary files can be found at https://figshare.com/articles/dataset/Supplementary_material_docx/24942498.
